# Supplementary material for: Qutrit toric code and parafermions in trapped ions
Source: Nat Commun. 2025 Jul 8;16:6301. doi: 10.1038/s41467-025-61391-z (PMC12238524; doi:10.1038/s41467-025-61391-z)
Supplement: Supplementary file 1 — Supplementary Information [file 41467_2025_61391_MOESM1_ESM.pdf]

SUPPLEMENTARY INFORMATION

Supplementary Methods

This section details the construction of primitive gates for manipulating $\mathbb{Z}_3$ qutrits, utilizing the native gate set of Quantinuum's H2-series devices [59, 60]. A key consideration in circuit design is the number of two-qubit ZZPhase gates (the native entangling gate) required in each decomposition, as this directly influences the implementation cost. We encode each qutrit degree of freedom into two qubits according to the mapping:

$$|0\rangle_{\text{qutrit}} := |00\rangle\,, \quad |1\rangle_{\text{qutrit}} := |10\rangle\,, \quad |2\rangle_{\text{qutrit}} := |11\rangle\,, \quad |\text{nc}\rangle := |01\rangle \tag{1}$$

The non-computational state $|\text{nc}\rangle$ is used to herald errors that cause the two encoding qubits to fall outside the qutrit space. Given that we have following single- and two-qubit gates natively available on the device,

$$U_{1q}(\theta,\phi) = e^{-i(\cos\pi\phi X + \sin\pi\phi Y)\frac{\theta}{2}} := \text{—}\boxed{(\theta,\phi)}\text{—}, \quad R_Z(\theta) = e^{-\frac{1}{2}i\pi\theta Z}, \tag{2}$$

$$\text{ZZPhase}(\theta) = e^{-\frac{1}{2}i\pi\theta(Z\otimes Z)}. \tag{3}$$

The action of the qutrit $\mathcal{Z}$, defined in (1), on the encoded space can be implemented efficiently using two single-qubit gates.

$$\mathcal{Z} := \quad (\text{circuit}) \quad = \bar{\omega}\ \text{diag}(1,\ \omega,\ \omega,\ \bar{\omega}) \tag{4}$$

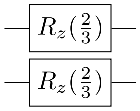

Importantly the action of $\mathcal{Z}$ on $|\text{nc}\rangle$ is trivial up to the phase. In contrast, implementing $\mathcal{X}$ requires two CNOT gates, or a single ZZPhase gate.

$$\mathcal{X} := \quad (\text{circuit}) \quad = \quad (\text{circuit}) \quad = \begin{pmatrix} 0 & 0 & 0 & 1 \\ 1 & 0 & 0 & 0 \\ 0 & 0 & 1 & 0 \\ 0 & 1 & 0 & 0 \end{pmatrix} \tag{5}$$

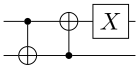

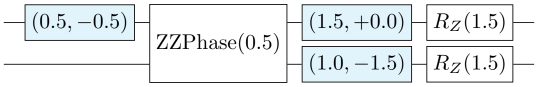

Similarly, the charge conjugation gate $\mathcal{C}$ can also be implemented using only a single ZZPhase gate.

$$\mathcal{C} := \quad (\text{circuit}) \quad = \quad (\text{circuit}) \quad = \begin{pmatrix} 1 & 0 & 0 & 0 \\ 0 & 0 & 0 & 1 \\ 0 & 0 & -1 & 0 \\ 0 & 1 & 0 & 0 \end{pmatrix} \tag{6}$$

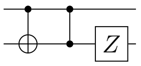

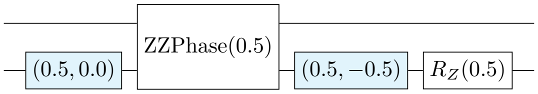

Another primitive gate for our purposes is the qutrit Fourier transform, denoted by $\mathcal{H}$, which acts on computational states as follows:

$$\mathcal{H}\,|i\rangle_{\text{qutrit}} = \frac{1}{\sqrt{3}}(|0\rangle_{\text{qutrit}} + \omega^i\,|1\rangle_{\text{qutrit}} + \omega^{2i}\,|2\rangle_{\text{qutrit}}), \tag{7}$$

while leaving $|\text{nc}\rangle$ unchanged. The native implementation of $\mathcal{H}$ requires three ZZPhase gates.

To construct $\text{C}\mathcal{X}$, we begin by implementing $\text{C}\mathcal{Z}$, which utilizes four Controlled-R_Z gates. Applying the $\text{C}\mathcal{Z}$ gate to a target qutrit transformed by $\mathcal{H}$, results in the required $\text{C}\mathcal{X}$ gate.

$$\text{C}\mathcal{X} := \quad (\text{circuit}) \tag{8}$$

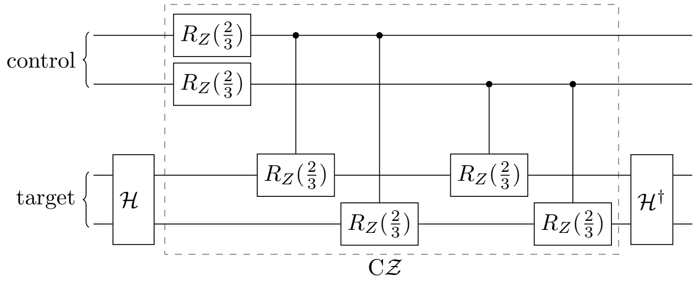

We observe that the action of $\mathcal{H}$ on the $|0\rangle_{\text{qutrit}} = |00\rangle$ results in the superposition $\frac{1}{\sqrt{3}}(|00\rangle + |10\rangle + |11\rangle)$. We can consider $\mathcal{H}|00\rangle$ as a state preparation procedure that can be implemented using only a single ZZPhase gate [61].

$\mathcal{H}|00\rangle =$

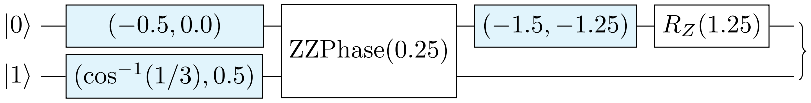

$\frac{1}{\sqrt{3}}(|00\rangle + |10\rangle + |11\rangle)$ (9)

Preparing the $\mathbb{Z}_3$ ground state on a 6×4 lattice using Quantinuum's H2-series native gateset requires 251 two-qubit gates for final destructive measurements in the $\mathcal{Z}$-basis and 189 two-qubit gates for measurements in the $\mathcal{X}$-basis. The circuit depth is 113 for $\mathcal{Z}$-basis measurements and 106 for $\mathcal{X}$-basis measurements.

Supplementary Note 1: Fidelity Bounds

1. $\mathbb{Z}_3$ Ground State

The projector onto the logical $|00\rangle$ ground state can be written as a product of projectors

$$|00\rangle\langle 00| = PQ \tag{10}$$

with

$$\begin{aligned} P &= \prod_{\substack{x\,\in\,\text{type-A}\\ \text{plaquettes}}} \Pi^1_{A_x} \\ Q &= \left(\prod_{\substack{z\,\in\,\text{type-B}\\ \text{plaquettes}}} \Pi^1_{B_z} \right) \Pi^1_{\mathcal{Z}-\text{hori}} \Pi^1_{\mathcal{Z}-\text{vert}}. \end{aligned} \tag{11}$$

Here, x and z run over all plaquettes of the given type except one and the geometry of the logical operators $\Pi^1_{\mathcal{Z}-\text{hori}}$ and $\Pi^1_{\mathcal{Z}-\text{vert}}$ can be chosen arbitraily — each choice leads to a valid bound.

Since $[P, Q] = 0$, we can label a complete set of states by their eigenvalues with respect to P and Q (denoted p and q which are each 0 or 1) such that we can decompose the density matrix of the prepared state as a convex combination

$$\begin{aligned} \rho &= \sum c_{pq}\rho_{pq} \\ &= c_{00}\rho_{00} + c_{01}\rho_{01} + c_{10}\rho_{10} + c_{11}\rho_{11}. \end{aligned} \tag{12}$$

Here, c_{11} is the target state fidelity that we are seeking to bound. Furthermore, we have

$$\begin{aligned} \text{Tr}[\rho P] &= c_{10} + c_{11} \\ \text{Tr}[\rho Q] &= c_{01} + c_{11} \\ 1 &= c_{00} + c_{01} + c_{10} + c_{11} \end{aligned} \tag{13}$$

from which we conclude our lower bound

$$\begin{aligned} c_{11} &= \text{Tr}[\rho P] + \text{Tr}[\rho Q] - 1 + c_{00} \\ &\geq \text{Tr}[\rho P] + \text{Tr}[\rho Q] - 1. \end{aligned} \tag{14}$$

On the other hand, we have as an upper bound

$$c_{11} \leq \min\{\text{Tr}[\rho P], \text{Tr}[\rho Q]\}. \tag{15}$$

For the $6 \times 4 = 24$ qutrit system, we have measured

$$\begin{aligned} \text{Tr}[\rho P] &= 0.75(2) \\ \text{Tr}[\rho Q] &= 0.68(3) \end{aligned} \tag{16}$$

for the global projectors. From this, the bound for the global fidelity and fidelity per qutrit

$$\begin{aligned} \langle 00|\,\rho\,|00\rangle &\in [0.42(4), 0.68(3)] \\ \langle 00|\,\rho\,|00\rangle^{1/24} &\in [0.965(3), 0.984(2)] \end{aligned} \tag{17}$$

follows. Employing SPAM error correction, as discussed in Supplementary Note 2, we obtain improved fidelity bounds:

$$\begin{aligned} \langle 00|\,\rho\,|00\rangle_{\text{SPAM error mitigated}} &\in [0.53(3), 0.73(3)] \\ \langle 00|\,\rho\,|00\rangle^{1/24}_{\text{SPAM error mitigated}} &\in [0.974(3), 0.987(1)]. \end{aligned} \tag{18}$$

2. Topological Qutrit States

The exact same argument is used to bound the fidelity of topological qutrits encoded in the entangled states of two pairs of charge conjugation defects. For example, in the case where the post-measurement state of the ancilla is $|0\rangle$, the state $|\mathbf{11}\rangle + |e\bar{e}\rangle + |\bar{e}e\rangle$ is prepared. In this case, P is the projector onto the $+1$ eigenspace of the operator denoted by $X_{\text{ent}}X_{\text{ent}}^{\dagger}$, where X_{ent} changes the internal states according to:

$$X_{\text{ent}} = |\mathbf{1}\rangle\langle e| + |e\rangle\langle\bar{e}| + |\bar{e}\rangle\langle\mathbf{1}|\,. \tag{19}$$

Note that $X_{\text{ent}}X_{\text{ent}}^{\dagger} = Z_L$, as defined in Sec. II D, and the gray loop in Fig. 5b depicts the concrete implementation of this operator in terms of $\mathcal{Z}$ and $\mathcal{Z}^{\dagger}$. Similarly, Q is the projector onto the $+1$ eigenspace of the $Z_{\text{ent}}Z_{\text{ent}}$ operator (as depicted by the orange loop in Fig. 5b), and Z_{ent} acts like:

$$Z_{\text{ent}} = |\mathbf{1}\rangle\langle\mathbf{1}| + \omega\,|e\rangle\langle e| + \bar{\omega}\,|\bar{e}\rangle\langle\bar{e}|\,. \tag{20}$$

Moreover, $X_{\text{ent}}X_{\text{ent}}^{\dagger}$ distinguishes the three topological qutrit states with eigenvalues of $+1$, ω, and $\bar{\omega}$. The expectation values of the gray and orange string loops are measured to obtain the values for the topological qutrit stabilizers $X_{\text{ent}}X_{\text{ent}}^{\dagger}$ and $Z_{\text{ent}}Z_{\text{ent}}$. We measure the following values:

Ancilla outcome	State of defect pairs	$(\Pi^{1}_{X_{\text{ent}}X^{\dagger}_{\text{ent}}}, \Pi^{\omega}_{X_{\text{ent}}X^{\dagger}_{\text{ent}}}, \Pi^{\bar{\omega}}_{X_{\text{ent}}X^{\dagger}_{\text{ent}}})$	$(\Pi^{1}_{Z_{\text{ent}}Z_{\text{ent}}}, \Pi^{\omega}_{Z_{\text{ent}}Z_{\text{ent}}}, \Pi^{\bar{\omega}}_{Z_{\text{ent}}Z_{\text{ent}}})$
0	$\lvert\phi_1\rangle = \lvert\mathbf{11}\rangle + \lvert e\bar{e}\rangle + \lvert\bar{e}_1e_2\rangle$	(0.92(3), 0.07(3), 0.01(1))	(0.80(4), 0.09(3), 0.11(3))
1	$\lvert\phi_\omega\rangle = \lvert\mathbf{11}\rangle + \omega\lvert e\bar{e}\rangle + \bar{\omega}\lvert\bar{e}_1e_2\rangle$	(0.009(9), 0.94(2), 0.05(2))	(0.75(4), 0.13(3), 0.12(3))
2	$\lvert\phi_{\bar{\omega}}\rangle = \lvert\mathbf{11}\rangle + \bar{\omega}\lvert e\bar{e}\rangle + \omega\lvert\bar{e}_1e_2\rangle$	(0.06(2), 0.02(1), 0.92(3))	(0.68(5), 0.11(3), 0.20(4))

Supplementary Table 1. **Measurement outcomes for the logical readout of the three different basis states of two charge-conjugation defect pairs.**

Repeating the argument above for each of the three post-measurement states and of the ancilla corresponding topological qutrit states $|\phi_1\rangle, |\phi_\omega\rangle$ and $|\phi_{\bar{\omega}}\rangle$, we obtain the bounds:

$$\begin{aligned}
\mathrm{Tr}[\langle\phi_1|\,\rho\,|\phi_1\rangle] &\in [0.72(5), 0.80(4)] && \text{if ancilla in state } |0\rangle\\
\mathrm{Tr}[\langle\phi_\omega|\,\rho\,|\phi_\omega\rangle] &\in [0.70(5), 0.75(4)] && \text{if ancilla in state } |1\rangle\\
\mathrm{Tr}[\langle\phi_{\bar{\omega}}|\,\rho\,|\phi_{\bar{\omega}}\rangle] &\in [0.60(6), 0.68(5)] && \text{if ancilla in state } |2\rangle\,.
\end{aligned} \tag{21}$$

Applying SPAM error mitigation, we again obtain improved fidelities:

$$\begin{aligned}
\mathrm{Tr}[\langle\phi_1|\,\rho\,|\phi_1\rangle]_{\text{SPAM error mitigated}} &\in [0.76(4), 0.83(4)] && \text{if ancilla in state } |0\rangle\\
\mathrm{Tr}[\langle\phi_\omega|\,\rho\,|\phi_\omega\rangle]_{\text{SPAM error mitigated}} &\in [0.73(5), 0.77(4)] && \text{if ancilla in state } |1\rangle\\
\mathrm{Tr}[\langle\phi_{\bar{\omega}}|\,\rho\,|\phi_{\bar{\omega}}\rangle]_{\text{SPAM error mitigated}} &\in [0.63(5), 0.70(5)] && \text{if ancilla in state } |2\rangle\,.
\end{aligned} \tag{22}$$

Each of these fidelities is significantly larger than the maximum fidelity achievable with a classical mixture.

Supplementary Note 2: Ground State Preparation Data Analysis

This appendix presents additional findings regarding the preparation of the $\mathbb{Z}_3$ ground state. We show the expectation values for the prepared ground state on a 6×2 and 4×4 lattice (cf. Fig. 1(a,b)). These values were computed by discarding heralded shots, similar to the data presented in Fig. 2(c). For these lattice sizes, we find the fidelity per qutrit/site $f := \langle 00|_L \, \rho \, |00\rangle_L^{1/n_{\text{sites}}}$, as discussed in Supplementary Note 1, to be:

$$
\begin{aligned}
f &\in [0.959(3), 0.978(2)], \quad \text{for } 6 \times 2, \\
f &\in [0.972(2), 0.985(1)], \quad \text{for } 4 \times 4.
\end{aligned}
$$

We observe a slightly lower per-qutrit fidelity on the 6×2 lattice compared to the 4×4 lattice. This difference might be attributed to the constrained geometry of the 6×2 lattice. This forces the unitary preparation protocol to order the plaquette preparation in a more chain-like manner, impacting circuit depth.

Figure 1(c) illustrates the effect on energy densities when heralded shots are not discarded. While a slight decrease in energy densities is observed, the overall impact is relatively small.

To evaluate the fidelity of the prepared state independently of the quality of qubit measurements, it is crucial to consider the expectation values of stabilizer projectors after employing measurement error mitigation. State preparation and measurement (SPAM) error mitigation accounts for state preparation and readout errors. In practice, since measurement errors significantly outweigh state preparation errors, we implement SPAM error mitigation in a simplified form by constructing the measurement error transition matrix based on Quantinuum's H2-1 prior device characterization parameters. Specifically, we use $p(\text{measure } 0|\text{qubit is } 1) = 2.37e-3$ and $p(\text{measure } 1|\text{qubit is } 0) = 0.82e-3$. The inverse transition matrix is then applied to each qubit by writing the raw probability distribution of measurement outcomes as a matrix product state of bond dimension n_{shots}.

In Fig. 1d we show the data while employing only SPAM error mitigation. In contrast, Fig. 1e displays the values where, in addition to SPAM error mitigation we discard heralded shots based on qutrit measuments. We find that both approaches, independently, contribute to an improved fidelity of the prepared state.

Since the depth of the unitary state preparation circuit is not uniform across all qutrits/qubits, we introduce a barrier at the end of the circuit before final measurements. This compiler command ensures that the system reaches the full $\mathbb{Z}_3$ ground state wavefunction before measurements collapse it into a product state. In Fig. 2(a,b), we contrast the impact of barrier insertion on the state fidelity. While there is a slight decrease in quality, the overall impact is negligible. Furthermore, by optimizing the state preparation for circuit depth, we can slightly improve the overall fidelity (cf. Fig. 2c).This optimization involves parallelizing gate operations not at the plaquette level but at the individual control-$\mathcal{X}$ gate level.

We observe similar behavior for the 6×4 lattice in terms of improving fidelities when discarding heralding shots and applying SPAM mitigation (see Fig. 3).

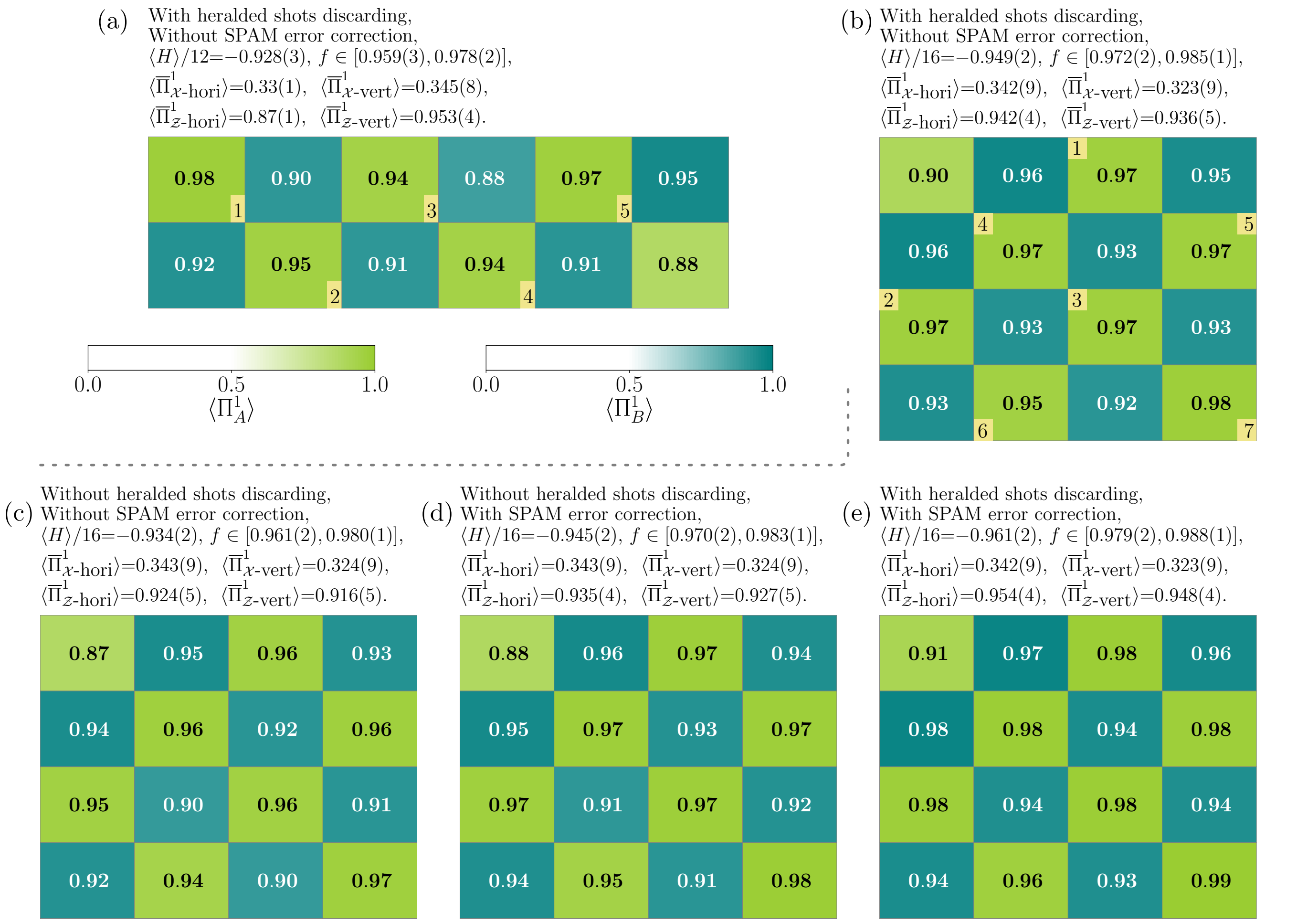

Supplementary FIG. 1. **Ground state energy densities.** Expectation values for stabilizers Π^1_A and Π^1_B on a torus. (a,b) We present preparation data for 6×2 and 4×4 lattices. For each type-A plaquette, a small square indicates the location of the control qutrit used in its preparation. The number within the square denotes the order in which the plaquettes were prepared. We discard heralded shots where qutrits are measured outside the qutrit space, while no other error mitigation technique is employed. (c,d,e) We compare the impact of heralded shot discarding and SPAM error mitigation on the expectation values of energy densities for the same 4x4 lattice as in (b)

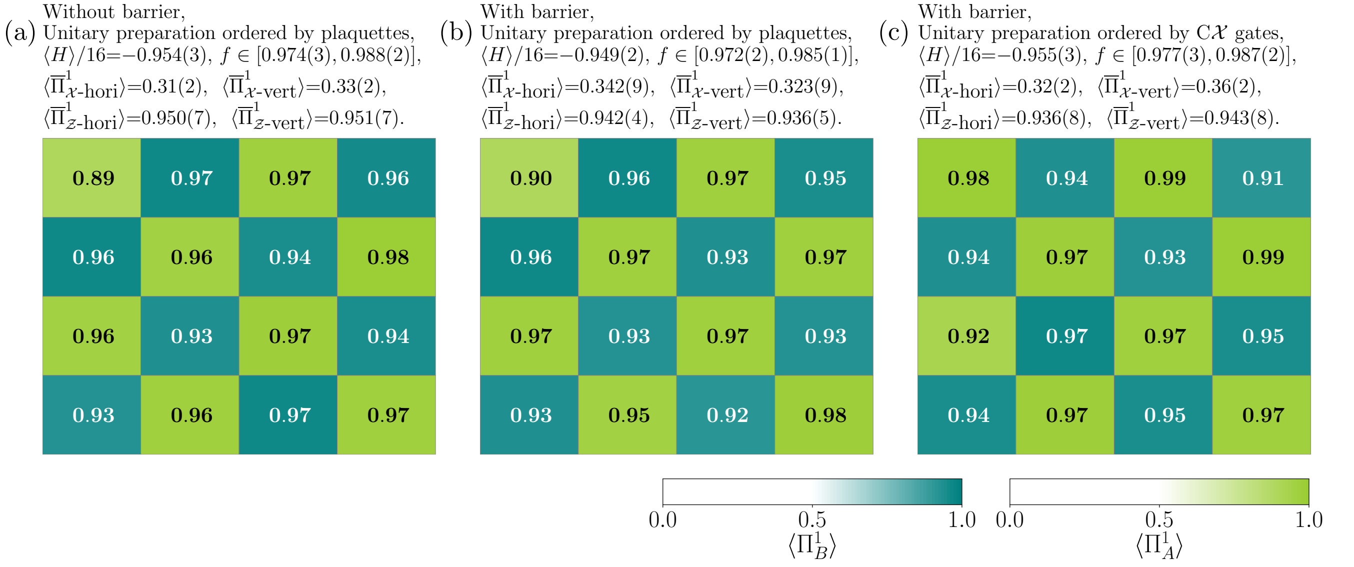

Supplementary FIG. 2. **Barrier insertion and gate ordering at the control-$\mathcal{X}$ level for state preparation.** (a) Presents preparation data for the ground state without a barrier before inal measurements of qutrits. (b) This shows preparation data with a barrier, identical to Fig. 1b and included for completeness. (c) We demonstrate preparation data optimized for circuit depth at the C-$\mathcal{X}$ gate level, rather than at the plaquette level.

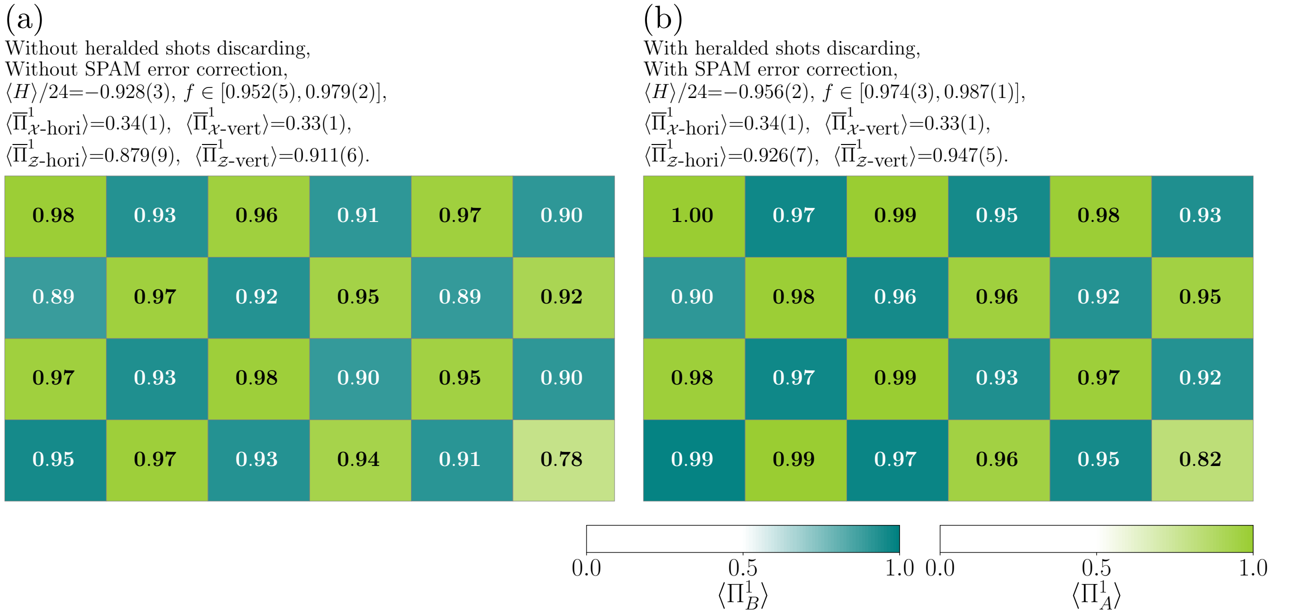

Supplementary FIG. 3. **Ground state energy densities for 6×4 lattice.** Expectation values for stabilizers Π^1_A and Π^1_B. (a) We do not discard heralded shots where qutrits are measured outside the qutrit space, and SPAM error mitigation is applied. (b) We discard heralded shots and employ SPAM error mitigation.

Supplementary Note 3: Topological qutrit on a 6×2 Lattice

This appendix presents a detailed investigation of entanglement transfer on a smaller 6×2 lattice. The reduced hardware costs associated with this smaller lattice allowed us to conduct a more comprehensive study compared to the larger 6×4 lattice.

We begin by preparing the ground state and then apply the circuits U_1 and U_2 to create two pairs of defects (Fig. 4(a,b)). The definitions of the transformed stabilizers are given in Fig. 4c. The resulting expectation values of the stabilizers are shown in Fig. 5a. We then initialize an ancilla qutrit 'a' in the state $\frac{1}{\sqrt{3}}(|0_a\rangle + |1_a\rangle + |2_a\rangle)$ and use it as a control to apply a control-$\mathcal{Z}_1$ gate. This entangles the stabilizers A_2 and A_5, resulting in the following state including the ancilla: $(|0_a\rangle\,|\mathbf{1}\mathbf{1}\rangle + |1_a\rangle\,|e\bar{e}\rangle + |2_a\rangle\,|\bar{e}e\rangle)$. Fig. 5b shows the resulting stabilizer values. The fact that stabilizers A_2 and A_5 are locally in a completely mixed state is demonstrated by their values, which are approximately 1/3.

By further applying $\mathcal{Z}_2$ and $\mathcal{Z}_3^\dagger$ (cf. Fig. 9c), we effectively shift the position of one end of the charge pair. This alters the state of defect pair 2, resulting in a joint state of: $(|0_a\rangle\,|\mathbf{1}\mathbf{1}\rangle\,|\mathbf{1}_2\rangle + |1_a\rangle\,|e\bar{e}\rangle\,|e_2\rangle + |2_a\rangle\,|\bar{e}e\rangle\,|\bar{e}_2\rangle)$. Next, we apply $\mathcal{Z}_4^\dagger$ and $\mathcal{Z}_5$ (as shown in Fig. 9d) to also entangle defect pair 1. By applying the operator $\mathcal{Z}_6^\dagger$, we fuse the charge anyon back with its partner as it completes a loop around the torus (Fig. 4e). This leaves the two defect pairs entangled with the ancilla, resulting in the following state:

$$|0_a\rangle\,|\mathbf{1}_1\mathbf{1}_2\rangle + |1_a\rangle\,|e_1\bar{e}_2\rangle + |2_a\rangle\,|\bar{e}_1 e_2\rangle.$$

The ancilla qutrit can be decoupled by measurement in the $\mathcal{X}$ basis. Depending on the measurement outcome, we are left with three distinct topological qutrit state and get the following values for the expectation values of stabilizers projectors:

Ancilla outcome	State of defect pairs	$\Pi^1_{A_0}$	$\Pi^1_{A_1}$	$\Pi^1_{A_0A_1}$	$(\Pi^1_{X_{\rm ent}X^\dagger_{\rm ent}}, \Pi^\omega_{X_{\rm ent}X^\dagger_{\rm ent}}, \Pi^{\bar\omega}_{X_{\rm ent}X^\dagger_{\rm ent}})$
0	$\|\mathbf{1}_1\mathbf{1}_2\rangle + \|e_1\bar{e}_2\rangle + \|\bar{e}_1 e_2\rangle$	0.31(3)<br>0.31(3)*	0.33(3)<br>0.33(3)*	0.83(2)<br>0.86(2)*	(0.92(2), 0.05(1), 0.03(1))<br>(0.94(2), 0.04(1), 0.017(8))*
1	$\|\mathbf{1}_1\mathbf{1}_2\rangle + \omega\|e_1\bar{e}_2\rangle + \bar\omega\|\bar{e}_1 e_2\rangle$	0.31(3)<br>0.31(3)*	0.27(3)<br>0.27(3)*	0.89(2)<br>0.92(2)*	(0.017(9), 0.94(2), 0.04(1))<br>(0.008(6), 0.96(1), 0.03(1))*
2	$\|\mathbf{1}_1\mathbf{1}_2\rangle + \bar\omega\|e_1\bar{e}_2\rangle + \omega\|\bar{e}_1 e_2\rangle$	0.31(3)<br>0.31(3)*	0.32(3)<br>0.32(3)*	0.82(3)<br>0.84(2)*	(0.06(2), 0.07(2), 0.87(2))<br>(0.05(1), 0.06(2), 0.89(2))*

Supplementary Table 2. **Measurement data for the topological qutrit on the 6×2 lattice.**

Numbers marked with an asterisk (*) were calculated using SPAM error mitigation, as outlined in Supplementary Note 2. The internal states of defect pairs are characterized by A_0 and A_1. While the projectors $\Pi^1_{A_0}$ and $\Pi^1_{A_1}$ have values close to 1/3, indicating a locally mixed state, the joint projector $\Pi^1_{A_0A_1}$ exhibits a high value for each ancilla outcome, suggesting a globally entangled state. The action of the gray string operator (illustrated in Figure 5e, with $\mathcal{Z}_1\mathcal{Z}_2\mathcal{Z}_3^\dagger\mathcal{Z}_4^\dagger\mathcal{Z}_5\mathcal{Z}_6^\dagger$) has the same effect as applying the logical $X_{\rm ent}X^\dagger_{\rm ent}$ stabilizer to the internal states of the topological qutrit. $X_{\rm ent}X^\dagger_{\rm ent}$ attains its maximum value in distinct sectors for each qutrit state, enabling their identification.

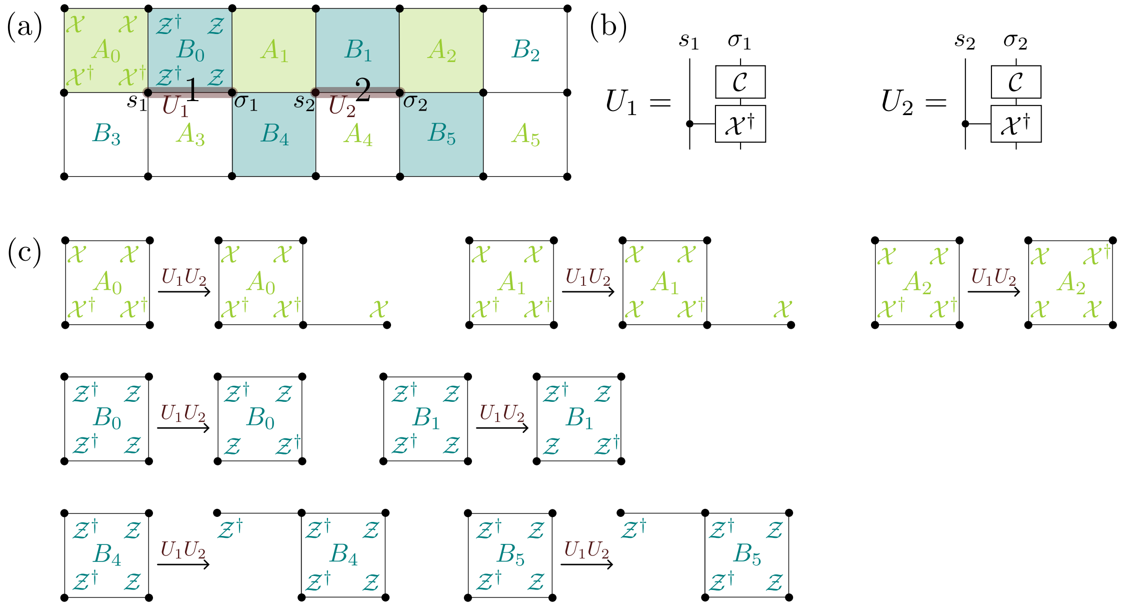

Supplementary FIG. 4. **CC defect pairs** Two CC defect pairs, labeled 1 and 2, are created on a 6×2 lattice. Defect pair 1 is generated by applying the unitary operator U_1 to qutrits s_1 and σ_1. Defect pair 2 is created by applying the unitary operator U_2 to qutrits s_2 and σ_2. The colored plaquettes highlight stabilizers that are non-trivially transformed under the action of U_1U_2. (b) The circuit constructions of the unitary operators U_1 and U_2 are shown. (c) Transformed stabilizers under the combined action of U_1U_2. The stabilizers A_0 and B_4 define the endpoints of defect pair 1, while stabilizers A_1 and B_5 define the endpoints of defect pair 2.

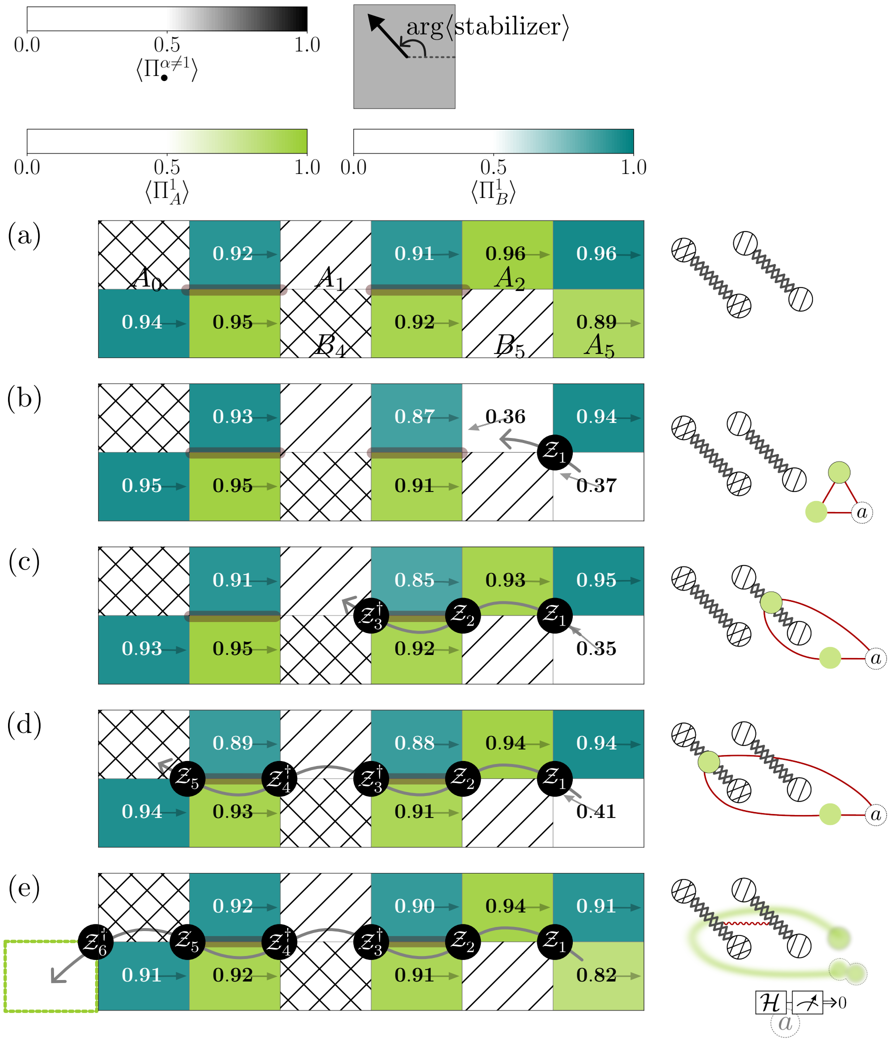

Supplementary FIG. 5. **Entanglement transfer to pairs of charge conjugation defects on a 6×2 lattice** We prepare the $\mathbb{Z}_3$ ground state and then create two pairs of charge conjugation defects. (a-e) The left-hand side displays data representing the expectation values of stabilizer projectors during different stages of entanglement transfer. The right-hand side provides a schematic representation to illustrate the corresponding state. Maximum error in estimating expectation value is 0.023.

Supplementary Note 4: Defects in the $\mathbb{Z}_3$ TC

The automorphism group of the $\mathbb{Z}_3$ TC is $\mathbb{Z}_2^2$, generated by two electromagnetic dualities $e \leftrightarrow m$ and $e \leftrightarrow \bar{m}$. Their product is charge conjugation $e \leftrightarrow \bar{e}$ and $m \leftrightarrow \bar{m}$. The quantum dimension of the em duality defects are $\sqrt{3}$ and charge conjugation is 3.

3. *Electro-Magnetic Duality Defects*

In the rotated toric code, we can create *em*-duality defects by measuring $\mathcal{Y} = \mathcal{X}\mathcal{Z}$ along a line and then applying some feed-forward correction based on the measurement outcomes.

Measuring $\mathcal{Y}$ on a single vertex fuses the $\mathcal{X}$ and $\mathcal{Z}$ stabilizers with support on that site together in the following way:

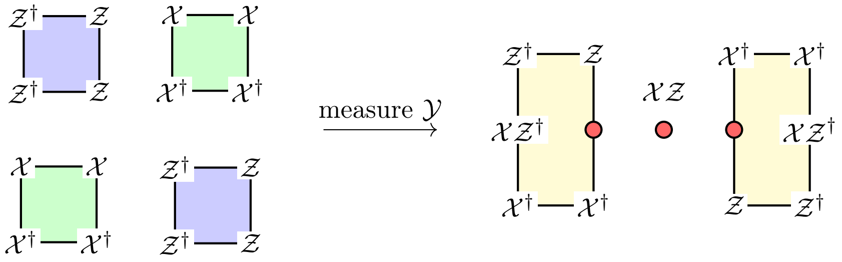

(23)

Additionally, there is a nonlocal stabilizer spanning both defect endpoints:

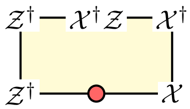

In this example, since we have created the minimum length defect line, this nonlocal stabilizer is the same weight as the endpoint stabilizers. As we grow the defect line, however, this defect will grow as well, so that it always connects the two endpoints.

Using these stabilizers, we can verify that crossing the defect line (here, it consists of the single measured vertex) transmutes an e to an m anyon. Consider a e anyon passing through the defect from top to bottom; in order to satisfy both defect stabilizers, the string must change from $\mathcal{Z}$ to $\mathcal{X}^\dagger$, meaning it becomes an m anyon on the other side. To keep the overall state neutral, the defect must have absorbed an $e\bar{m}$ anyon—this is captured by the fact the nonlocal stabilizer now has an eigenvalue of $\omega^\dagger$ rather than 1.

4. *Charge Conjugation Defects*

We present here unitary and measurement-based circuits for creating such an open charge conjugation line. A more detailed discussion can be found in [47], in addition to a general derivation of anyon and defect ribbons for generic quantum doubles.

First, we give an example circuit for creating a pair of charge conjugation defects. The shaded plaquettes indicate the location of the defects created by this circuit.

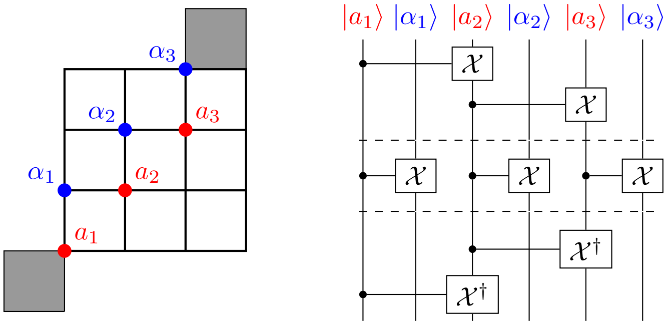

(24)

We now present a derivation of this circuit. Consider the $\mathbb{Z}_3$ toric code with the following stabilizers (on alternating plaquettes, as in the main text):

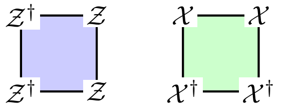

Charge conjugation is a global symmetry of the $\mathbb{Z}_3$ toric code; acting with $\mathcal{C}$ on every degree of freedom preserves the ground state manifold and the braiding properties of any excitations. We can create a closed charge conjugation boundary by acting with charge conjugation in some finite region A of the lattice (see Fig. 6a). This doesn't affect the stabilizers that are completely contained in the region A or the region $A^\perp$—only the stabilizers along the boundary ∂A will get modified:

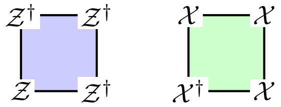

When an e or m anyon crosses this boundary, it will get mapped to its conjugate particle. We can see this by considering what strings are valid (i.e. only create excitations at their ends) crossing the boundary. Away from the boundary, the usual e-string ($\mathcal{Z}$ along a diagonal) commutes with the unmodified stabilizers. When crossing the boundary, we have to have to switch to an $\mathcal{Z}^\dagger$ since the e-string now overlaps with two $\mathcal{X}^\dagger$ operators on the boundary X plaquette. In this way, the e particle has been turned into an $\bar{e}$ (see Fig. 6a).

In some sense, however, all we have done is change the anyon labeling conventions inside region A. There will be no physically measurable effects of the $e \to \bar{m}$ transformation, since if we bring this anyon to fuse back with its partner, it will cross the charge conjugation boundary a second time, returning to its original state (see Fig. 6a again).

More nontrivially, we can consider creating an open charge conjugation line, with two charge conjugation point defects at either end. These defects cannot be created by acting with $\mathcal{C}$ in a bulk region. If such a pair of defects are present in the system, we can actually end up with measurable effects. For instance, if we have an open charge conjugation line, we can braid an e around one endpoint; since it crosses the defect line only once, when it reaches its $\bar{e}$ partner, they will fuse back to a single e (see Fig. 6b). The residual $\bar{e}$ needed to preserve overall neutrality of the state is now stored nonlocally in the internal Hilbert space of the charge conjugation defect line.

Our goal is to find a circuit capable of creating such open charge conjugation defect lines. Essentially, we want a circuit which has the same action as a bulk charge conjugation symmetry membrane on the $\mathbb{Z}_3$ toric code ground state, but which is localized to the boundary of the membrane. This boundary circuit can be straightforwardly truncated to yield an creation operator for a pair of charge conjugation defects.

To derive and explain Eq. 24, note that $\prod \mathcal{C}$ is a global $\mathbb{Z}_2$ symmetry of the $\mathbb{Z}_3$ paramagnet $\prod_v (|0\rangle + |1\rangle + |2\rangle)$, along with the $\mathbb{Z}_3$ symmetry $\prod \mathcal{X}$. After applying the $\mathbb{Z}_3$ Kramers-Wannier map [24], the $\mathbb{Z}_3$ paramagnet is mapped to the $\mathbb{Z}_3$ toric code—we say that these two phases are dual to each other. Charge conjugation acts trivially in a finite region of the $\mathbb{Z}_3$ paramagnet—therefore, all the key properties of the charge conjugation symmetry membrane on the $\mathbb{Z}_3$ toric code are captured by the gauging map itself. By 'pushing' the membrane from the $\mathbb{Z}_3$ toric code through the $\mathbb{Z}_3$ gauging map back to the paramagnet, we can simplify the bulk operator to some action purely at the membrane boundary.

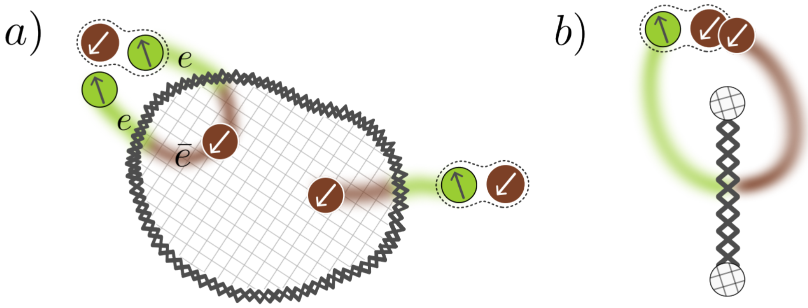

Supplementary FIG. 6. **Closed vs. open charge conjugation defect lines:** a) Applying $\prod \mathcal{C}$ in the shaded region creates a closed charge conjugation boundary; any anyon that crosses the boundary will be transmuted to its conjugate. Given the region is closed, any closed anyon loop must intersect the boundary an even number of times, meaning there are no measurable fusion results to determine the presence of such a boundary. b) An open charge conjugation has point defects living at its ends. Anyons can braid nontrivially with these defects, leading to nontrivial fusion outcomes.

The $\mathbb{Z}_3$ gauging map $\mathsf{KW}_{\mathbb{Z}_3}$ takes paramagnetic degrees of freedom (green plaquettes) to the vertex degrees of freedom in the following way:

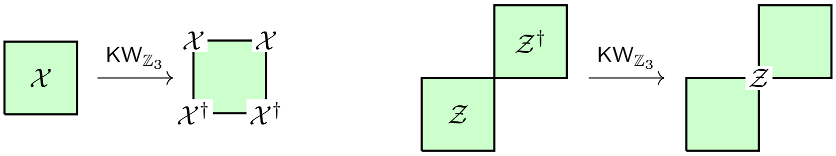

(25)

These expressions fully specify the action of the gauging map on $\mathcal{C}$, as

$$\mathcal{C} = |0\rangle\langle 0| + \mathcal{X}|1\rangle\langle 1| + \mathcal{X}^\dagger |2\rangle\langle 2| = \sum_{n=0}^{2} \mathcal{X}^n T_n \tag{26}$$

where $T_n = |n\rangle\langle n| = \frac{1}{3}(1 + \omega^{-n}\mathcal{Z} + \omega^n \mathcal{Z}^\dagger)$. Using this decomposition, we can apply the gauging transformation rules to find the following identities:

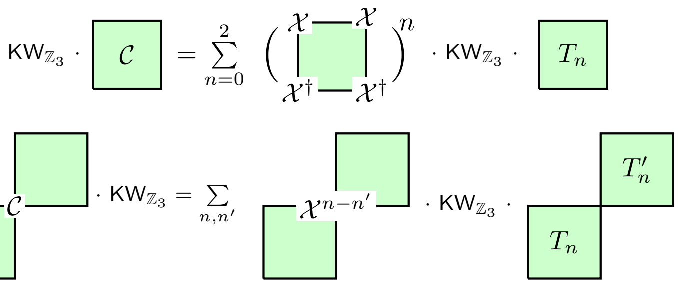

(27)

We see that a single-site charge conjugation operator prior to the gauging map is equivalent to vertex operator after the gauging map, controlled on the pre-gauged state. Similarly, conjugation acting from the toric code side is equivalent to a product of C$\mathcal{X}$, controlled on the neighboring pre-gauged sites. Figure 7 illustrates how the above identities can be used to reduce a bulk charge conjugation defect acting on the $\mathbb{Z}_3$ toric code to a boundary circuit plus a bulk charge conjugation action on the trivial paramagnet. However, the paramagnet is invariant under even a finite charge conjugation membrane, and so these gates can be ignored. Only the boundary circuit remains, given by:

$$\prod_{s\in\gamma} \mathrm{C}\mathcal{X}^{\eta(v_s)}_{s\to v_s} \tag{28}$$

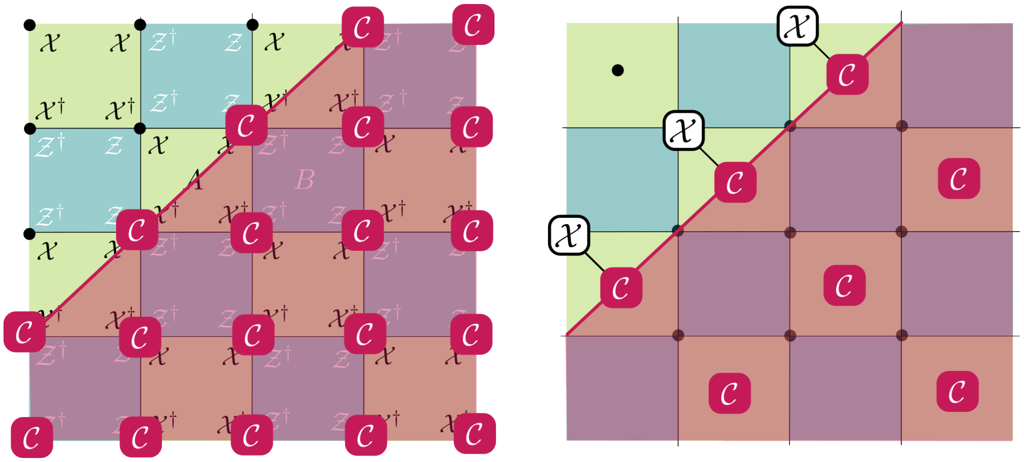

Supplementary FIG. 7. **A bulk charge conjugation membrane is equivalent to some boundary action:** Left: A bulk charge conjugation symmetry membrane acting on the toric code degrees of freedom, which creates a charge conjugation defect boundary. Right: Via the properties of the gauging map, the membrane is equivalent to a charge conjugation membrane on the dual paramagnetic degrees of freedom, plus a finite-depth circuit of control gates at the boundary, with the paramagnetic degrees of freedom as controls and the toric code degrees of freedom as targets. The charge conjugation gates act trivially on the paramagnet and can be ignored.

where γ is the desired charge conjugation boundary, s is a paramagnetic degree of freedom lying on the boundary γ, v_s is the neighboring toric code degree of freedom just outside the boundary (see Fig. 7), and $\eta(e) = +1(-1)$ if v_s lies on the left (right) side of the plaquette corresponding to s.

We have partially derived the example unitary circuit. However, the above finite-depth circuit acts on both pre-gauged sites and gauged vertices; in the experiment, we only have access to the gauged toric code degrees of freedom. To circumvent this issue, we use the one-dimensional counterpart of the $\mathbb{Z}_3$ gauging map to access the necessary pre-gauged sites. This is valid due to the presence of a 1-form symmetry in the $\mathbb{Z}_3$ toric code; this 1-form symmetry acts like a global symmetry along a chosen 1D path, making the application of the 1D gauging map valid. The 1D gauging map essentially uncovers the pre-gauged degrees of freedom needed to act as controls for the finite-depth portion of the circuit.

The 1D gauging map can be implemented unitarily via a linear-depth, sequential circuit:

$$\mathsf{KW}^{1\mathrm{D};\gamma}_{\mathbb{Z}_3} = \prod_{i\in\gamma} C\mathcal{X}_{i,i+1} \tag{29}$$

This completes the derivation of the charge conjugation defect circuit: (1) apply the 1D gauging map to reveal needed pre-gauged degrees of freedom, (2) use the derived finite-depth boundary circuit, (3) apply the (un)-gauging map to go back to toric code degreees of freedom everywhere. Written explicitly, we have:

$$U^{\gamma}_{c.c.} = \mathsf{KW}^{1\mathrm{D};\gamma\dagger}_{\mathbb{Z}_3} \left(\prod_{s\in\gamma} \mathrm{C}\mathcal{X}^{\eta(v_s)}_{s\to v_s} \cdot \mathcal{C}_{v_s} \right) \mathsf{KW}^{1\mathrm{D};\gamma}_{\mathbb{Z}_3} \tag{30}$$

Supplementary Note 5: Coherently moving the end of a charge conjugation defect

First, we define what coherently moving the end of a CC defect pair means operationally. Given a CC defect pair ending on site A and site B, we have coherently moved the end of a CC defect pair at site B if there is no remnant particle and the stabilizers at site B return to their original form. Therefore, to coherently move one end of a CC defect from site B to site C, we can apply another ribbon operator with ends at B and C, and ensure that the two CC defects at site B is in the vacuum fusion channel.

Next, we define the internal states of a CC defect. Consider the example of CC defect in Fig. 9, upon applying the unitary U (Fig. 9b), the A_0 stabilizer at the start of the ribbon becomes non-local, and the B_5 stabilizer at the end of the ribbon becomes non-local (Fig. 9c). The non-local e and m anyon determines the internal states of a CC defect, so we should label the internal state of a CC defect as $\left|e^a, m^b\right\rangle$. If $a = 0$ ($b = 0$), then it indicates that the non-local e (m) stabilizer is not violated. This is a basis for the possible internal states of a CC defect pair, so the quantum dimension of one CC defect is $\sqrt{9} = 3$, as expected.

Now, we discuss the reason why applying U again is operationally equivalent to coherently moving the start of a CC defect. Initially, applying U creates a CC defect pair in the internal state of $\left|e^0, m^0\right\rangle$. The CC defect unitary circuit satisfy $U^2 = 1$; i.e. if we apply U again, we would have coherently moved an end of a CC defect pair and returned to the vacuum. However, after crossing an m anyon through the CC defect, the internal state becomes $\left|e^0, m\right\rangle$. After applying another U, we are essentially overlaying another CC defect pair with internal state of $\left|e^0, m^0\right\rangle$. At the start, the two ends fuse to vacuum, whereas at the end, the two ends does not fuse to vacuum, and hence we see a remnant particle of m.

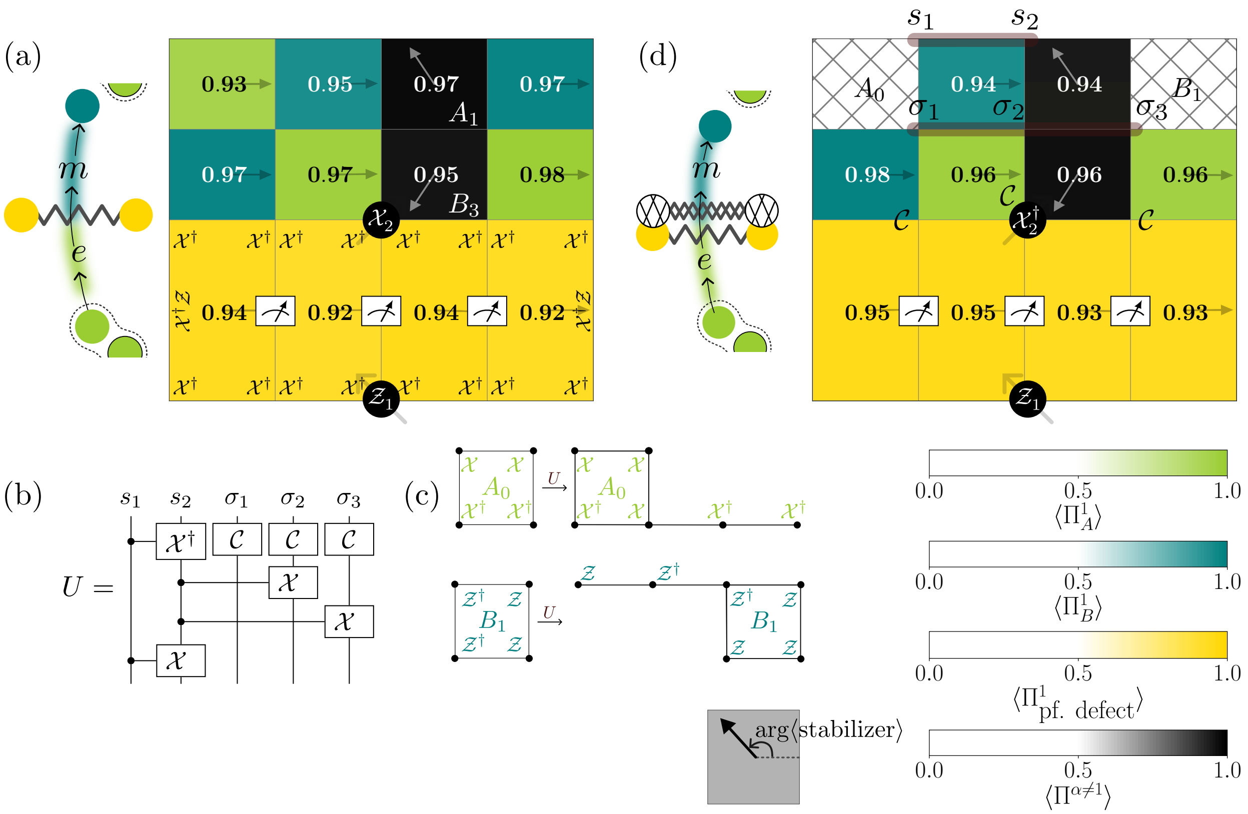

Supplementary FIG. 8. **Fusion of charge conjugation and parafermion defects** (a) We prepare the ground state and create a parafermion defect line by measuring three qutrits. Depending on the measurement outcomes, we apply feed-forward operations to initialize the internal states of the parafermion defect line. We act with $\mathcal{Z}_1\mathcal{X}_2$ on qutrits on two sides of the defect line. This creates an $\bar{e}-m$ anyon pair on plaquettes $A_1 - B_3$. (b) Circuit construction of a unitary to create a CC defect line. (c) Transformation of stabilizers at the endpoints of the CC defect line after applying the unitary U. (d) Starting with the ground state containing a parafermion defect line, we apply the unitary U from (b) to create a CC defect line. We then deform the CC defect line by applying $\mathcal{C}$ gates. This effectively results in a conjugated parafermion line, as demonstrated by the fact that the correct string to create an $\bar{e}-m$ anyon is now $\mathcal{Z}_1\mathcal{X}_2^\dagger$.

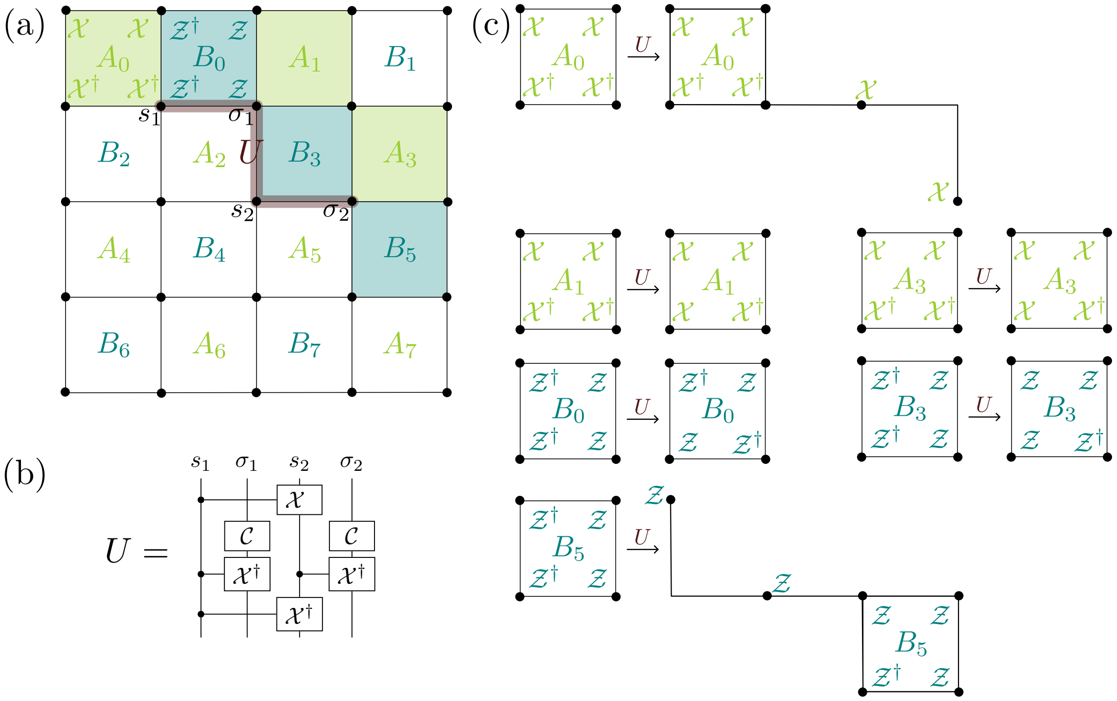

Supplementary FIG. 9. **Charge conjugation defect pair on a** 4×4 **lattice** (a) Square lattice on torus. A charge conjugation defect pair is created by a unitary action U along the string of qutrits labeled s_1, σ_1, s_2, and σ_2. Stabilizers that transform non-trivially under the action of U are highlighted by coloring the corresponding plaquettes. (b) Circuit used to create the charge conjugation defect pair. (c) Non-trivial transformations of A_p and B_p stabilizers under the unitary action. Stabilizers that remain invariant under unitary action are omitted. The stabilizers A_0 and B_5 define the endpoints of the defect pair and characterize its internal state.

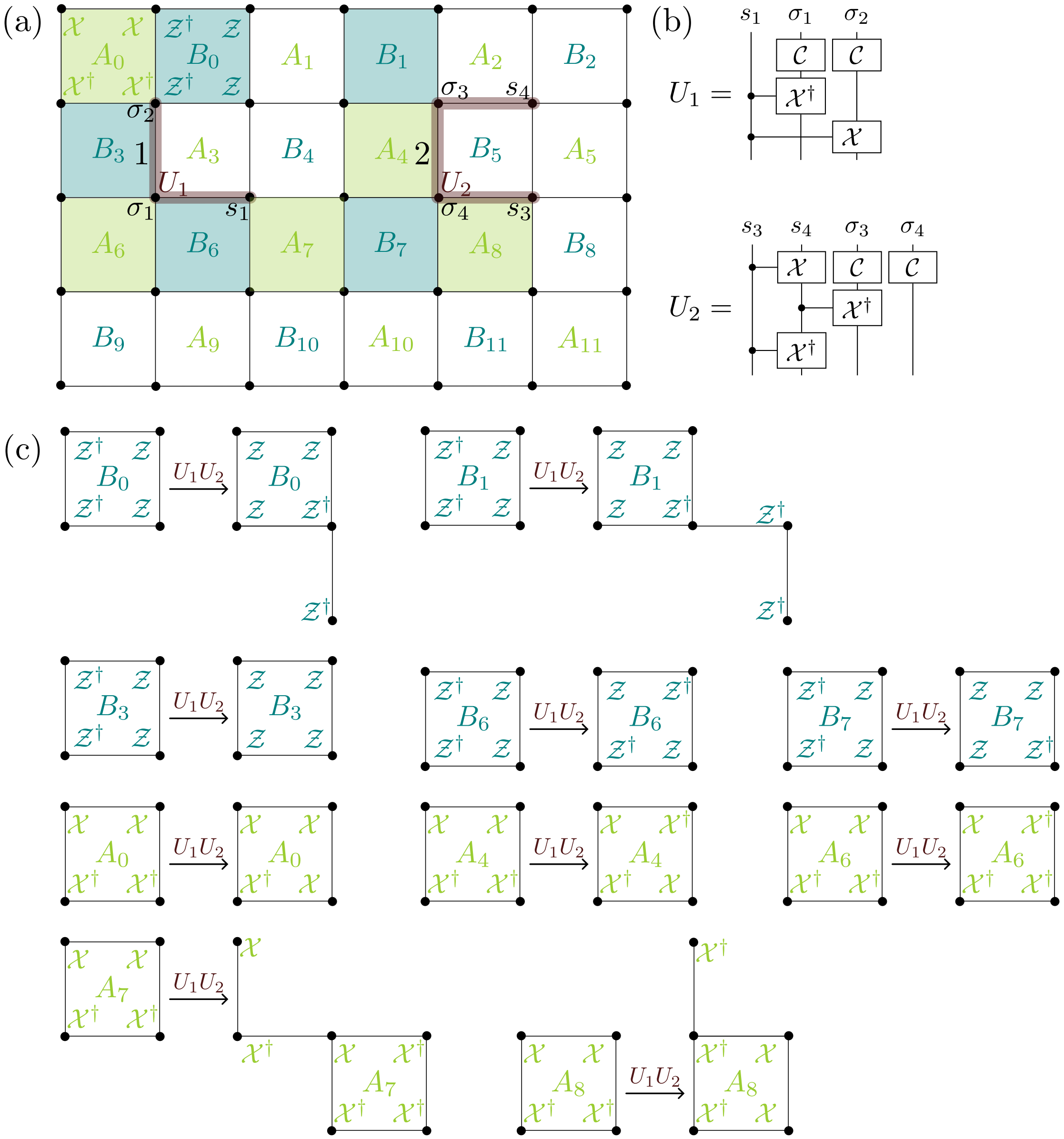

Supplementary FIG. 10. **Two charge conjugation defect pairs on a 6×4 lattice** (a) Two charge conjugation defect pairs, labeled 1 and 2, are created. Defect pair 1 is generated by applying the unitary U_1 to qutrits labeled s_1, σ_1, and s_2. Defect pair 2 is created by applying the unitary U_2 to qutrits labeled s_3, s_4, σ_3, and σ_4. Colored plaquettes indicate non-trivially transformed stabilizers. (b) Circuit construction of unitaries U_1 and U_2. (c) Transformation of stabilziers under the action U_1U_2. Stabilizers B_0 and A_7 define the endpoints of defect pair 1, while stabilizers B_1 and A_8 define the endpoints of defect pair 2.
